# “If somebody had told me I’d feel like I do now, I wouldn’t have believed them…” older adults’ experiences of the BELL trial: a qualitative study

**DOI:** 10.1186/s12877-022-03174-5

**Published:** 2022-06-03

**Authors:** Neil J. Meigh, Alexandra R. Davidson, Justin W. L. Keogh, Wayne Hing

**Affiliations:** 1 Faculty of Health Sciences and Medicine, Bond University, Bond Institute of Health & Sport, Gold Coast, QLD 4226 Australia; 2 Sports Performance Research Centre New Zealand, AUT University, Auckland, New Zealand; 3 Kasturba Medical College, Manipal Academy of Higher Education Mangalore, Manipal, Karnataka India

**Keywords:** Older adults, Kettlebell, Group exercise, Physical activity, Healthy ageing, Arthritis, Low back pain

## Abstract

**Objectives:**

This study examined older adults’ experiences of participating in the Ballistic Exercise of the Lower Limb (BELL) trial, involving 12-weeks of group-based hardstyle kettlebell training.

**Methods:**

In the BELL trial, 28 insufficiently active older adults (15 women, 13 men, 59–79 years) completed six weeks of face-to-face group training, and six weeks of home-based training. In-depth semi-structured interviews were audio recorded, transcribed, and inductively coded, with themes constructed from patterns of shared meaning.

**Results:**

Four higher-order themes were developed that reflect older adults’ experiences participating in a group-exercise program of hardstyle kettlebell training. These included: (1) “It’s one of the best things we’ve done”—enjoying the physical and psychosocial benefits, (2) “It’s improved it tremendously!”—change in a long-term health condition, (3) “It put me on a better course”—overcoming challenges, and (4) “I wasn’t just a number”—feeling part of a group/community.

**Discussion:**

Findings highlight the perceived physical and psychological benefits of older adults participating in hardstyle group kettlebell training, and the value attributed to being part of an age-matched community of like-minded people engaged in group exercise. Implications for program design and delivery, and future research, are discussed.

**Supplementary Information:**

The online version contains supplementary material available at 10.1186/s12877-022-03174-5.

Life expectancy is increasing in many countries worldwide [1]. Recent estimates suggest that approximately 16% of the Australian population are aged 65 or over, with this number projected to increase to 21–23% by 2066 [2]. An ageing population and shift in demographic composition, increases the social and economic burden of ill-health, chronic disability, and disease, with the number of healthy years lost to disability, also increasing [3]. The term ‘older adult’ is defined by the United Nations, Cochrane, and numerous reviews, as ≥ 60 years of age [4–8]. Regular physical activity has consistently been found to be protective for older adults facing declines in the domains of physical and cognitive function, self-reported health and vitality, mental health, and mortality [9–19], with vigorous exercise performed for longer periods providing the greatest benefits [20, 21]. Older adults however, are the least active age group in society [22], with over 70% of Australian adults aged over 65, insufficiently active [2], defined as not meeting the Australian Physical Activity Guidelines for older adults [23]. Exercise is the primary means of preventing or reversing the age-related loss of physiological reserve, with frailty accelerated by physical inactivity [24]. Lack of engagement in regular exercise and physical activity is due, in part, to sedentary behaviours being strongly linked with positive reinforcement [7], thus meaning it is difficult to increase engagement in physical activity at a community level. A significant challenge, therefore, is to identify and implement efficacious initiatives that increase long-term physical activity behaviours among older adults.

The health benefits of regular exercise are well documented [25, 26], with strength and endurance being strong indicators of health and predictive of mortality [27]. Extending the years spent functionally independent, allows older adults to continue pursuing enjoyable activities and improve their quality of life, and reduces the risk of depression and loneliness [28]. There has been growing research interest in older adults’ engagement with physical activity initiatives [29], with reported factors including perceived benefits, social connectedness, and being part of a group [30–32]. Additionally, older adults have reported that trying new activities, having a positive attitude, and engaging with life and self-improvement are important components for ageing well [33]. Combined, these factors make an activity meaningful. Group exercise is particularly rewarding [34], and older adults returning to the gym have described a renewed enthusiasm for it [35].

‘Resistance training’ uses the external resistance of machines and free weights, such as dumbbells and barbells, to improve muscular fitness. A kettlebell is a type of free weight resembling a cannonball with a handle, with which a wide range of resistance exercises may be performed. Kettlebell training, popularised by Pavel Tsatsouline in the 1990s and early 2000s [36], has been shown to improve the physical function of older adults with Parkinson’s disease [37], and body composition, strength, and pulmonary function in older females with sarcopenia [38]. Data from younger adults also suggests the potential to improve dynamic balance [39], further adding to its interest in healthcare as an attractive intervention for an ageing population. Findings from the Ballistic Exercise of the Lower Limb (BELL) trial, clearly support its use for improving grip strength, and measures of health-related physical fitness [40].

Healthcare providers have an important role in promoting and prescribing physical activity and exercise as part of routine care [41], with higher rates of adherence to regular exercise associated with improvements in self-reported health and well-being [42]. As a novel and previously untested mode of exercise for otherwise healthy community-dwelling older adults, the BELL pragmatic controlled trial was a replication of a community-based initiative, conducted to test the effectiveness of group-based hardstyle kettlebell training to promote healthy ageing to insufficiently active older adults. In this study, we explored experiences of the trial participants, to answer the question: was engagement sufficiently high and positive, to warrant a recommendation that group-based kettlebell training initiatives be used to promote healthy ageing in the community? The results highlight some of the challenges and opportunities of using kettlebells to promote physical activity among community-dwelling older adults, and may be used to inform program delivery and future research.

## Methods

The study was approved by the Bond University Human Research Ethics Committee (NM03279), with the design, conduct, and reporting of this study adhering to the Consolidated criteria for reporting qualitative research (COREQ) guidelines [43]. The study involved participants from the BELL trial. Full details of the design and conduct of the BELL trial have been published elsewhere [40]. All research activities were conducted in accordance with relevant guidelines and regulations, in accordance with the Declaration of Helsinki. Written informed consent was obtained from all participants. The BELL trial was pre-registered on the Australian New Zealand Clinical Trials Registry (ACTRN12619001177145).

### Participants

Thirty-two apparently healthy, but insufficiently active older adults (59–79 years), were recruited to complete 12-weeks of moderate to high-intensity hardstyle kettlebell training as a part of the BELL trial. Three participants withdrew during the control period: medical condition (*n* = 1), injury (*n* = 1) and no longer able to attend (*n* = 1). Twenty-nine participants commenced training, with 28 completing six weeks or more. All participants in the present study completed at least half (≥ six weeks) of the training intervention (13 men and 15 women, 68.8 ± 4.6 years). One male, who withdrew due to work in the second week of the intervention, was not invited for interview. All remaining participants in the BELL trial were interviewed, including four participants who withdrew: substance abuse and mental health (*n* = 1), low back pain (*n* = 1), viral infection (*n* = 1), uncontrolled hypertension: GP requested (*n* = 1). All participants were Caucasian. Comorbid health conditions at baseline included: obesity, controlled hypertension, depression, diabetes, peripheral neuropathy, osteoporosis, sarcopenia, cancer, osteoarthritis (hip and knee), persistent non-specific low back pain, Ankylosing spondylitis, hypercholesterolemia, immunosuppression, migraines, alcohol dependency and poor sleep.

### Study setting

Participants attended 45-min group-exercise classes three-times weekly (Mon, Wed, Fri), and completed prescribed home exercise twice-weekly (Tue & Thur). Face-to-face group classes were conducted by the first author for six weeks, then remotely thereafter due to COVID-19 restrictions. The first author is a 45-year-old male kettlebell instructor and physiotherapist. Several strategies were used to enhance participant engagement, including frequent individual and group encouragement (recognition of overcoming challenges, extraordinary effort, and achieving a ‘personal best’), daily communication via a private Facebook group and email (to foster a spirit of group support, accountability, camaraderie, and healthy competition), and encouraging post-workout gatherings (on-site café) to promote social connection outside of class. Additionally, the instructor took part in the training where possible, to be seen as an active part of the group experience.

The BELL trial utilised a 12-week control period, followed by a 12-week intervention,, with the participants fully aware of the goals and objectives of the research. This provided the researcher with a unique opportunity to interact with the participants, and for thoughtful and reflexive engagement in the qualitative data which were subsequently collected. One in three participants who commenced training in February 2020, continued to train together beyond the intervention period (June 2020), and were still training weekly at the time of pre-print publication. The first author remained in regular contact with the group to the time of publication.

### Procedures

Data collection took place 6^th^—15^th^ May 2020, commencing three working days after completion of the planned intervention period. A study timeline and changes made due to the covid-19 pandemic, are reported elsewhere [40]. Participants were invited to take part in an in-depth semi-structured interview, for up to an hour. Interviews were held via Zoom due to COVID-19 restrictions, with the participants at home. Interviews were conducted by the first author who, at the time of the study, was a doctoral student responsible for the BELL trial. Interviews were digitally recorded and transcribed using Trint transcription software [44]. All participants in the study were interviewed separately, and no eligible participants declined to be interviewed. There were no repeat interviews. Given the relationship between the interviewer and participants, interviews were not piloted, and field notes were not taken. Transcriptions were checked against the audio recordings line by line, with errors corrected as necessary before being imported to NVivo 12 software [45] for coding and thematic analysis. Transcripts were not returned to participants for comment, however authenticity of quotes was maintained [46].

An interview guide (see Table 1) was used as a framework however, questions were posed flexibly in an open-ended way, allowing key experiences to be discussed. The interview guide was not pilot tested. Participants were given the opportunity to talk about topics which were not part of the guide, allowing space for unexpected insights to be explored [47]. Interviews were informed by the participant’s responses to a series of quantitative rating-scale questionnaires, which had been completed via Survey Monkey following cessation of the training period. Questionnaire responses highlighted areas of interest, allowing the interviewer to ask targeted questions to gain a better insight of meaning, and discuss key topics. Questionnaires were developed based upon the first author’s disciplinary expertise, and experience working with the participants during the training period. Overarching topics were, i) participants’ experience training with kettlebells, ii) perceived positive and beneficial effects, and iii) negative and undesirable effects. Questionnaires are available as supplementary data. Interviews with each trial participant continued until the researcher felt that data saturation had been reached, with no new insights emerging [48]. At this point, the interview was concluded.

**Table 1:** Interview guide

	Questions
1	What were your motivations for wanting to try kettlebell training?
2	How has your experience been different to what you had expected?
3	Tell me about the things you enjoyed the most
4	How do you see yourself using kettlebells now that the trial has finished?
5	What is the most noticeable physical change you have seen, if any?
6	What positive effects have you noticed, if any?
7	If you were to do the trial again, how would you approach it differently?
8	Tell me about the things you did not like about the training?
10	What has been of most value to you, either as a participant in the trial or from using kettlebells?
11	Has anything surprised you?
12	What are you most pleased with having achieved?
13	How has kettlebell training compared to other forms of exercise you have done in the past?
14	How would you change the training so that it better suited you?

### Philosophical underpinnings and data analysis

This qualitative study, which was subsumed within the BELL trial project, is underpinned by a philosophy of critical realism (CR), a useful approach for analysing social challenges and developing solutions for change [49]. To examine older adults’ experience participating in group kettlebell training, data from in-depth, semi-structured interviews, were thematically analysed, with codes and themes developed inductively using reflexive thematic analysis [50].

The data analysis was led by the first author, who had conducted the training intervention with the study participants. Familiar with the lived experience of the participants, the first author began by immersing himself in the data, through reading and re-reading transcriptions of the audio recordings. Consistent with CR ontology, initial data analysis and coding began with a search for ‘demi-regularities’ at the empirical level (the participants description of events) and identification of tendencies (trends or patterns acting as causal tendencies) [51]. The first round of coding utilised a combination of coding categories, such as emotion (participants’ feelings e.g., “fun”), value (values, attitudes and beliefs e.g., beliefs of effect or experiential value), narrative (participants’ stories e.g., social interaction), evaluation (assigning judgement e.g., increased self-confidence), and process coding (observable activity e.g., walking more), as well as theming data (meaning e.g., grocery shopping has become easier) [52]. Codes and initial themes from collated data were developed iteratively, both semantic and latent, until the entire data set was complete, resulting in the construction of 21 broad themes. The focus was to identify patterns of shared meaning within the participant’s accounts of their experience in the trial. Inductive thematic saturation was achieved, with no new codes or themes emerging [48].

The first and second authors met to review and further analyse the codes developed by the first author, to refine, define, and name higher-order themes. The second author is a doctoral student whose study exclusively utilises qualitative research methods. She had not been involved with any other part of the BELL trial and did not meet the participants. Second round coding methods included pattern (e.g., identifying trends and relationship), focused (e.g., most frequent), axial (e.g., identifying core categories and dimensions) and selective coding (e.g., connections to form a storyline) [52]. Transcripts were reviewed to refine the themes, and to choose quotations to support interpretations.

Themes were subsequently reworked and redefined during the writing process. After the main findings had been identified through coding, abduction (theoretical redescription) was used to re-describe and raise the theoretical engagement beyond thick description, using general ideas about exercise and resistance training, community-based group programs with older adults, and kettlebell training [51]. Finally, retroduction (inference of causal tendencies) was used to describe the participants’ experiences [51]. To maintain a commitment to complexity, and highlight some of the challenges of delivering an exercise intervention to a diverse group of older individuals, the analysis includes exceptions to central themes and discussion of their potential impact [53]. Final higher-order themes are presented in the findings below.

## Results

Four themes were developed that describe how the participants perceived and experienced the BELL trial: (1) “It’s one of the best things we’ve done”—enjoying the physical and psychosocial benefits, (2) “It’s improved it tremendously!”—change in a long-term health condition, (3) “It put me on a better course”—overcoming challenges, (4) “I wasn’t just a number”—feeling part of a group/community. Pseudonyms are assigned, and sex has been changed in some cases, to protect participant anonymity.

### “It’s one of the best things we’ve done”: Enjoying the physical and psychosocial benefits

Participants described an improved sense of self-confidence from feeling fitter and stronger.

“I’ve got a pool, so I always keep a few 20 kilo bags of salt around the place. I throw that around now like a bottle of milk… our coffee table is really heavy and I just pick one end of it up [to vacuum underneath]… my wife won’t let me tie up the garbage bins with the bags [anymore], because when I tie them up now in a knot I tear the bag completely!” Samuel, 70-74 years old

Participants reported being more engaged in incidental physical activity, and less breathless with prolonged exercise, such as long walks and climbing hills. Participants were happy and excited to be re-engaging in physical activities which they had previously cut back, or stopped doing altogether, and being more active with their partners. Domestic activities, particularly those involving relatively heavy objects such as carrying shopping bags, moving furniture to clean, and gardening, were said to have become much easier.

Participants described feeling more energetic and motivated. They enjoyed having goals to aim for, and felt proud when they were able to reach and exceed them. Participants liked the discipline of having to be somewhere and do something every day, and appreciated that it was “fun”, with an opportunity to meet people and make new friends. Participants felt that the training had changed their appearance, describing muscles in their arms and legs has having more tone. They described how others in the group appeared to be walking taller, and family members had noticed that they were carrying themselves differently and doing more around the home.

“I’m walking, going out with the wife, doing things, and I’m confident in myself as I walk around that I’m not an ‘old’ person, I’m [an older person] that looks healthy and I’m quite enjoying that. …Having the opportunity to do something, seeing it through, finishing it, being able to challenge myself; I’m a different [man]” Alan, 70-74 years old.

Participants said they enjoyed learning something new. They liked that the training was challenging, the competitiveness of trying to better their own achievements, and comparing themselves to the performance of others in the group. Competition and goal-attainment gave a sense of accomplishment, and participants enjoyed being able to record and track their progress. Participants liked that training sessions were always different and said that not knowing what to expect each class had kept it interesting and made them feel engaged. Participants joked about “old” stereotypes and described the factors that had reinforced feelings of still being very physically capable: learning exercises they perceived to be too hard, being able to do things which they thought would be beyond their capacity, getting to the end of a hard training session, and attaining a new ‘personal best’.

Participants reported sleeping better and feeling healthier. Exploring their physical limits with heavy weights, and pushing themselves hard, had been empowering; it gave them the confidence to perform physical tasks that they had previously been anxious about, or avoided altogether. Several participants also described an improved balance, including one whose balance had been adversely affected by chemotherapy. This had increased their confidence during day-to-day tasks requiring greater balance, such as walking up and down stairs while carrying objects, and climbing in and out of a bath tub.

“Just having a shower, I used to get tired, and as for getting dressed, … I don’t have to hang on to something so I don’t fall over. … cutting my toenails, I never used to be able to do that by myself. …I used to tire out walking up a set of stairs, but now I can do that easily… It’s just been unbelievable the difference in myself. … for me to achieve what I’ve been doing being a smoker, well I just can’t be happier. It’s just unbelievable what I can do. … If somebody had told me I’d feel like I do now, I wouldn’t have believed them, … It’s just made me so much [more] enthused, like if I want to go across the road to get the paper, I wouldn’t ever think about jumping in the car now, I’d either hop on my bike or walk because you can do that now, … it’s one of the best things we’ve done… I feel so much stronger.” Peter, 70-74 years-old

Participants described being more flexible, and how activities such as rising from a low chair had become easier. These improvements in physical function made participants feel good about themselves, which had been reinforced when friends and family noticed the positive changes, or friends their own age told them they couldn’t or wouldn’t be able to do what they were doing.

“I’ve got a lounge chair at home which is relatively low, and I purposely hop up from that chair by not using [my] arms… I put my arms out in front of me and stand up from below normal seat height and I do that every time now and I haven’t got a problem with it. Before, I’d either have to push myself up, or turn over to one side and then stand up. I don’t do that anymore”. Thomas, 70-74 years old

### “It’s improved it tremendously!”: Change in long-term health condition

Many of the participants described how the training had positively influenced a long-term health condition, which had negatively affected their life or physical function. These conditions included hip and knee arthritis, persistent non-specific low back pain, osteoporosis, diabetes, peripheral neuropathy, cancer, ankylosing spondylitis, migraines, alcohol dependency, immunosuppression, obesity, hypertension, hypercholesterolemia, depression, and poor sleep.

All participants who said they had painful arthritic knees described a significant reduction in pain after the trial. In a few cases, participants said their symptoms had initially been irritated or worsened by some exercises, such as lunges, but all the participants who had been living with sore knees reported significant improvements in their arthritic knee pain and function at the end of the trial:

“Since I adjusted the exercises, I’ve been enjoying it because I don’t have the pain. If you’re enjoying doing it, it’s a motivation to do it… the pain abated, and I still got fit, so it was good”. Spencer, 65-69 years old

Participants either adapted an exercise so that it was more comfortable, or they chose to push through discomfort regardless. One participant with knee arthritis said he no longer had any pain.

“I can carry three loads of groceries up the stairs and… they’re quite steep, whereas before with my knee I couldn’t do it”. Abigail, 65-69 years old

Participants described significant reductions in other painful conditions. One participant, who consistently performed considerably more home exercise than required, had been living with frequent migraines, pain associated with an inflammatory arthropathy, frequently had insufficient sleep, and had a history of trips and falls. After the trial, she reported less frequent headaches, less pain, improved sleep, and better balance:

“My migraines, they’re less frequent and less severe… to me, wellbeing is not feeling sick with a headache or not having the same nagging pain in my hips… [my] overall well-being is much better… I used to have to go and have massages nearly every week, and I haven’t been once since I’ve been doing kettlebell [training]… I’m not constantly looking down to see that I’m not going to trip over… I’m not feeling as unsteady… I’ve had such a good response to it… I sleep like a baby” Janice, 60-64 years old.

Several participants had also been living with persistent low back pain. Except for one individual with a 50-year history of back pain who withdrew from the trial, everyone living with back pain described significant improvements in their symptoms and function.

“When I was getting out of bed before I started the trial, [I] always worried about [my] back going or something, but now I just can’t believe how much stronger my back is now. I’ll get up in the morning, get out of bed and there’s just not one bit of pain in there… It’s definitely one hundred percent better than what it was before I started the trial. It’s just unreal, I can’t believe it.” Robert, 70-74 years old

“I’m thrilled! I’m fit as a fiddle, I feel good, I sleep well, and I have no more back problems. I used to have once a month a physio come here to maintain my back [ ] I think I don’t need him [any more], I’m fine.” Georgina, 70-74 years old

Participants described feeling mentally buoyed by their participation in the trial. The profound positive psychological impact that engagement in the trial had, was most powerfully described by one participant who had been living with a mental health disorder for 20 years:

“This was the challenge I need… it’s given me the motivation to continue and that’s sort of saving me… this has been brilliant… I’m actually scared coming out the other side of this… I’m still in a bit of trouble…I have to keep going with this otherwise I’ll go back to where I was last year and not in a good place”. Katherine, 70-74 years old

### “It put me on a better course”: Overcoming challenges

Participants described the training as being harder than they had expected, at times uncomfortable even painful. Swinging the kettlebell overhead to perform a snatch was difficult to learn and created a sense of fear and apprehension among participants who found it difficult or impossible to press an 8 kg kettlebell overhead. But the participants persevered with the training, improved their technique, worried less, and even learnt to enjoy the some of the things they had found to be most challenging at the start.

“When you said we’d be swinging this thing above our head, everybody’s like ‘well I won’t be doing that’ and ‘that won’t be me’, and then when we all did it, it’s like, ‘we can do this, we’ll just keep on going’. That was good; that you got us to do something that I think we were all a little bit hesitant about, [but] by the end of it, that’s my favourite thing, I love it”. Irene, 60-64 years old

Participants found it was not easy to get up and down from the ground to perform a Turkish get-up, describing insufficient lower limb strength, not having enough flexibility, or having sore knees. Participants who really struggled with the Turkish get-up it, or said the lunging movements irritated their knees, found it demotivating. But they persevered and found ways to make it easier, so that they could do it. The Turkish get-up pattern was widely recognised as a floor transfer. Participants saw it as having more value than some of the other exercises because they could see how they were able to use it at home. One participant described how he had jumped onto a kitchen benchtop and performed a Turkish get-up to change a lightbulb. Participants described an increased sense of physical self-confidence having practiced and improved their ability to stand up from the ground.

“When we first had to do those … I thought I was slow as molasses. I had an ache and pain here and there and now I find myself using the Turkish get-up if I’m sitting on the floor”. Lucy, 65-69 years old

Delayed onset muscle soreness (DOMS) affected most participants, particularly in the first few weeks, sometimes occurring after exercises described as “easy”. Many participants were unfamiliar with the sensation of DOMS and unprepared for how it might impact them. In some cases, participants were reluctant to move much because of the discomfort, sufficiently concerned to describe it as “severe”. None of the participants allowed muscle soreness to prevent them from fully participating or allowing it to get in the way of their continued engagement with the group. Participants with a history of using resistance exercise were much less affected by muscle soreness and generally undeterred by it, and for some, muscle soreness was seen as positive, because it made them feel like they had done something which was “working”, and it brought back happy memories of being younger and more physically active. When aches and pains occurred, participants remained stoic and carried on regardless, describing a shared commitment to the trial and a determination to reach the end.

“[I thought to myself] that was too easy, am I doing it correctly? … how hard can it be, you’re just stepping up and down. I just kept going and I couldn’t move the next day. It was really bad [laughing]… It was so easy to do I thought this is nothing. It wasn’t until the next day, I thought holy cow! The [DOMS in my] calves on both legs was shocking, I could hardly move”. Iris, 65-69 years old

Participants living with pain commonly held maladaptive beliefs and behaviours relating to perceived risk of harm, believing they might hurt themselves if they pushed too hard, lifted too much, or did something which was too uncomfortable.

“The fear [that] people my age have about injury is paramount. We all like to be brave and like to think that we can do things, but sometimes either we don’t do as much as we should and can [do] for fear of hurting ourselves. We just need that extra encouragement to realise, yes, you can do this and it’s okay”. Kathy, 65-69 years old

Regardless of their concerns, participants continued to push themselves very hard. This was typified by ‘Ken’ who had previously been a competitive sportsman. Ken accumulated, by a very large margin, the highest training load volume in the group. He was so motivated and capable that he felt he was not being pushed hard enough: “at times, I just felt I was held back a bit with what I wanted to do”, and later went on to comfortably perform two-handed swings with a 40 kg kettlebell at home. But, after three decades of occupational health and safety training with his employer, Ken started the trial anxious that poor core strength and technique just leaning across the bed to pull up the doona (duvet) might hurt his back:

“I was really hesitant beforehand. I think I said to you very early, ‘aren’t we going to hurt our backs doing this?’ and you said ‘no’. … That’s been on my mind all the way through; at some stage I’m going to do my back, but I never did. … now there’re no inhibitions with what I’m doing, I’m confident … that I’m not going to hurt myself”. Ken, 70-74 years old

The training created numerous physical, psychological, and emotional challenges for the participants, to the extent that some thought about quitting in the early stages. However, no participants quit for any of these reasons, instead choosing to persevere and overcome them. A strong sub-theme, which overlapped with the participants’ enjoyment of the program, was the instructor’s role in facilitating this collective can-do mindset. Participants reported that the instructor’s personality, enthusiasm, leadership, encouragement, and happy, helpful demeanour, had been motivating and that was why they had continued to push themselves as hard as they had, often despite obvious challenges.

“I valued ‘you’ the most, because the time and effort that you put into all of us was awesome, and just the confidence that I had that you knew what you were talking about and that you cared about what we were doing and how we were doing it… I had a lot of confidence; that to me was the most important thing” Janice, 60-64 years old.

The participants’ attitude toward overcoming challenges was exemplified by Sean, who had experienced persistent shoulder pain for more than half of the trial. During the trial he was also diagnosed as iron deficient, told he had arthritic knees bad enough to warrant surgery, that a rotator cuff problem was the cause of his shoulder pain, and needed surgery to correct cataracts:

“I was starting to I think I was getting old and decrepit and demented, and that (training) picked me up out of that black hole… you got us all going; it’s your personality… that made me go harder than I would have… I was so buoyed because of the improvement in my physical health… It put me on a better course going into my older years that’s for sure.” Sean, 70-75 years old.

The spirit of the group was perhaps best displayed by Samuel who, in his 70 s, had volunteered to participate shortly after a life-changing conversation with his doctor. After his GP had questioned why he wanted to participant in the trial, Samuel told his doctor that he just wanted to be as healthy as he could be. Facing significant adversity, Samuel successfully completed the trial describing remarkable changes in his physical and mental health, all while performing swings at home with a 36 kg kettlebell.

### “I wasn’t just a number”: Feeling part of a group/community

The group dynamic was one of the strongest perceived benefits for participants. Being among people of a similar age, with varying abilities, provided participants with a rich and rewarding social connection. The camaraderie was fun and enjoyable, and allowed participants to support and encourage one another to succeed and push themselves harder than they would otherwise have done on their own. The group environment, where everyone was starting as a beginner, also gave participants the opportunity to try something new. Participants were more likely to engage in structured exercise if it was with others, and felt part of a positive, supportive, and encouraging community. Andrew, who described himself as a loner, said:

“In the group, I found that I was more open to being nicer and more inclusive with more people, so it really taught me that I can be part of the group and enjoy people.” Andrew, 65-69 years old

Men described being competitive more frequently than women, and competitiveness was regarded as beneficial, irrespective of how well someone was perceived to be doing in training. For example, those who were doing “better” typically enjoyed being able to help their partners when they were struggling, while those “chasing” the leaders, appreciated seeing someone their own age doing better than them, as it gave them something to aim for; “if they can do it, then so can I!” Participants especially enjoyed activities performed in pairs, as this allowed those who had formed social friendships outside of the gym to work together. The value of the group dynamic was typified by Sandy who, after a short period of absence due to back pain, was worried that others had progressed without her, and that she may not be accepted back into the group:

“They were all so happy to see me and it just made me feel that I wasn’t just a number; I was appreciated and that everyone cared how I was, and they didn’t [even] know me very well, … it just made me feel good, … I felt more positive about coming back, … [it] just made me realise how much I was enjoying it, … coming back, … made me realise how good I had been feeling, … I suddenly realised there were so many positive things that I’ve been experiencing that I’d overlooked and then when I got back, I realised, ah, this is what I was actually experiencing.” Sandy, 60-65 years old

The value of the social connection which the group provided, was most profoundly felt when face-to-face training was cancelled due to COVID-19 restrictions. Participants missed seeing the people they had formed new friendships with, and found it difficult to maintain the same level of motivation to train at home. This was somewhat mitigated by a private Facebook page, which allowed people to connect on a social level and share their progress, watch video recordings and live streams of training sessions, and take part in frequent ‘challenges’. The value of the social connection and support was perhaps most poignantly expressed by one participant whose partner was very unwell throughout the trial: “the contacts and friendships I’ve enjoyed from your trial have been and still are the most beneficial and a comfort to me”, Jennifer 65–69 years old.

## Discussion

The purpose of this study was to investigate the experiences of participants in the BELL pragmatic controlled trial. The aim of the BELL trial was to assess the effectiveness of three months moderate-to-high intensity group-based hardstyle kettlebell training, on grip strength and health-related physical fitness, in healthy but insufficiently active older adults. In this accompanying qualitative study, we sought to answer the question: was engagement sufficiently high and positive, to warrant a recommendation that group-based kettlebell training initiatives be used to promote healthy ageing in the community?

The results of this study offer some insight into the physical and psychosocial health benefits, and challenges, for older adults training with kettlebells. Sickness, medical procedures, and muscle soreness had negligible impact on rates of attendance and compliance, which remained very high. Participants perceived group training to be a friendly, supportive, and encouraging environment, where they could meet like-minded people of a similar age and make new friends. In the following sections we discuss the results in the context of existing literature, make recommendations for group-based community kettlebell programs for older adults, and consider directions for further investigation.

Many of the findings in the present study, echo those of the ‘GOAL’ trial [34], which involved community-dwelling older adults (≥ 65 years), performing group exercise for 50–60 min three times weekly, for three to six months. Similarities include: i) enjoyment and engagement fostered by social connection, ii) post-exercise interaction (an embedded component of both trials) enriched social connections, iii) self-perceptions of physical health and capacity were enhanced by group exercise, iv) high- and low-performing outliers had a preference to train with others of a similar physical capacity, v) training with similar-age peers was preferential to regular group exercise with mixed-age adults, and vi) dismay once the intervention ended. Notable differences, however, were that some of the participants in the present study said they had preferred training with people of the opposite sex, and the high- and low-performing outliers did not report any loss of affiliation with, or enjoyment of, the groups’ activities.

Enjoying the physical and psychological benefits. Participants described doing more around the home, and engaging in activities which they had previously found difficult, or had avoided altogether. Perceived benefits of feeling stronger and fitter, enhanced perceptions of physical appearance, and increased self-confidence, were consistent with previous studies [34, 54–57]. Improvements in cardiovascular fitness and strength are important outcomes for older adults, however, promoting the affective domains of fun, enjoyment, camaraderie, friendship, and community, are key to maximising the uptake of older adults into new programs of physical activity and exercise [58]. Consistent with Bredland et al. [59], some males expressed that they enjoyed being able to ‘flex their muscles’, and had felt more “useful” at home. Females spoke of their social relationships, the groups’ positive energy, having developed a habit of exercising, and changes in their physical capacity, previously identified as important resources for maintaining health [60].

Results from this study suggest that group-based kettlebell training, improved older adults’ perceptions of health and wellbeing, increased incidental activity, and enhanced their functional capacity to perform activities of daily living. Participants enjoyed the variety of exercises that kettlebell training provided, which is important for enjoyment and engagement [61, 62]. They liked the challenge of pushing themselves hard, and described the physical and psychological achievements as being empowering. Some participants even suggested their involvement had given them a sense of purpose.

### Change in a long-term health condition

Those with long-term health conditions, such as knee osteoarthritis and back pain, spoke of the training having disconfirmed maladaptive beliefs and behaviours relating to their condition. This is important, as these fears can be a barrier for older adults remaining active in later life [34, 63]. Pain and health conditions were not described as barriers to engagement. On the contrary, one male living with cancer frequently outperformed his younger counterparts, and a female with an inflammatory arthropathy, frequently recorded some of the highest daily training loads in the group.

Leveraging a social support network can enable someone who is living with pain, to participate in and be able to enjoy physical activity and exercise, despite their symptoms [64]. Thornton et al. [65] recommend clinicians lead by example, as this provides credibility and empathy for the challenges facing patients. This sentiment was echoed by participants in the present study, who said they felt encouraged and reassured seeing the instructor doing the same exercises, and also finding them hard. For people living with painful chronic health conditions, the influence of exercise on pain is equivocal, with mostly small to moderate effects [64]. For low back pain, exercise on its own has only small positive effects on disability, and limited ffects on coping [41]. Although the mediators and moderators are unknown, the experience of participants in this study, however, suggests a greater reduction in back pain than previous studies using exercise and resistance training [66, 67].

Participants who were living with long-term health conditions, described characteristics of grit and determination in overcoming the challenges of pain and unpleasant symptoms. Improved motivation and increased self-efficacy were reported to have come from the direction and guidance provided by the instructor, and the support and encouragement of peers. Exercise and physical activity in all its forms, can be used as a coping strategy for people living with painful conditions. Additionally, people living with pain seek opportunities to be active and want guidance from their healthcare provider [68]. Person-specific support can be greatly beneficial to help people make sense of their symptoms, and facilitate them engaging with regular exercise and meaningful physical activities [69]. There is considerable value in receiving accurate information and practical help from a trusted healthcare provider [70], because when advice is conflicting, people in pain can withdraw from social activities confused and anxious [71]. Results from this study, suggest that group-based kettlebell training with an appropriately experienced and enthusiastic instructor, can help enable people living with painful conditions to flexibility persist.

Overcoming challengesThe intervention was not however an optimal fit for all participants, with some asking to do more, and others saying they might have preferred a more moderate intensity and slower pace. This suggests a need to identify individuals likely to benefit from a different approach to program delivery, i.e., groups of a similar physical capacity, same sex, or one-on-one. Some participants expressed that they may have preferred to train with someone of a similar physical ability to themselves; a condition previously reported to undermine enjoyment and sense of connection to a group [34], but an effect not evident in the present study. Participants who struggled with physical limitations, did express feeling like they didn’t belong in the group. There was, however, no indication that this had reduced their enjoyment or engagement. Exercise has previously been reported as potentially off-putting if the intensity is perceived to be too high [72], and unappealing if too easy [73]. Training intensity in the BELL trial was often reported as “hard”, but the sense of achievement in completing it, was frequently described as rewarding. More evident among the males, and previously described by Bredland et al. [59], was competitiveness. Some males thrived on the challenge of doing more than others, some enjoyed the ‘chase’, and some would knowingly push themselves too hard.

### Feeling part of a group/community

Being part of a community of similar-age people and having a social network, was a strong theme, consistent with previous studies [34, 72]. These results align with a growing body of evidence which emphasises the importance of social interaction for older adults, in developing a positive attitude towards physical activity [74–76]. Social connection was most evident among participants who described having developed a personal bond with a training partner, which is a tenet of self-categorisation theory [34].

Participants described feeling supported by a competent, enthusiastic, and encouraging instructor who was motivating, and inspired confidence. The importance of the instructor in group exercise for older adults has previously been described [35, 77–79], where nurturing positive beliefs can improve participant self-efficacy, competence [80] and adherence [81]. The instructor’s personality, professionalism, and a humanised approach, have previously been identified as key factors in helping older people maintain adherence to programs in the long-term, as these can help participants feel cared for, and establish a sense of belonging [32]. In the moments when older people doubt their abilities and capacity, it is imperative that instructors allay these fears and instil a sense of confidence [35]. The results of this study, highlight the importance of an encouraging instructor who listens, participates in the training, and helps older adults to challenge themselves.

Echoing the work of Tulle and Palmer [82], participants in the present study were motivated to take part as an act of agency, to make a contribution to social change, and to challenge ageing stereotypes. Numerous motivators and barriers for older adults engaging with, and continuing in, programs of resistance training have been reported [72]. Feeling a part of a group is highly motivating [73, 79, 83], as this increases self-efficacy and feelings of well-being, decreases psychological stress [84], and provides a source of support, enjoyment, and belonging [31, 81, 85]. Older adults are typically less motivated to exercise at home [86], which was described by many participants in the BELL trial when group training was withdrawn due to COVID-19. However, the participants remained sufficiently motivated to maintain adherence rates over 90%, with everyone recording a ‘personal best’ on the final day of training. Previous studies have reported concerns regarding guidance and safety performing home-based exercises [87], but those issues were not expressed by the participants in the present study.

## Practical implications

Most Australian adults expect to receive advice about physical activity from a physiotherapist, and feel this is important [88]. Therapists who provide one-on-one interventions in their clinic to promote physical activity, double the likelihood of someone meeting the recommended physical activity guidelines, but these are only effective in the short-term and don’t appear to be effective for those living with chronic musculoskeletal conditions [89]. Similar reports of small short-term effects have been reported with group-based interventions, with no apparent difference between the type or frequency of intervention [90]. Community-based allied health therapists currently provide a range of exercise-based interventions to promote healthy ageing, based on programs such as the Otago Exercise Program for falls prevention [91], and Onero for promoting bone health [92]. Obtaining the perspective of community-based practitioners, around issues such as system-level barriers to providing physical activity and health-promoting programs for older adults, is essential [93].

The BELL trial intervention was a replication of a community-based program, which had been delivered within a physiotherapy private practice. Based upon the clinically significant effects of kettlebell training [40], and the qualitative results reported in this study, further research is warranted to gain a better understanding of the individual-level characteristics and system-level challenges which are currently limiting the delivery, utilisation, and effectiveness of healthy ageing programs in the community, and to better understand the factors which made the BELL trial successful. Pilates group exercise is widely uses in physiotherapy practice [94]. For older adults, the greatest effect of Pilates is to decrease the risk of falls, with only moderate improvements in strength and functionality [95]. Kettlebells are comparatively inexpensive, small, portable, and require much less floor space than a Pilates reformer. A lower cost of entry, and the potential for a larger number of participants per class, may make kettlebell classes a more attractive value proposition for community-based healthcare practices than other forms of group exercise requiring large, expensive pieces of equipment. Older adults are motivated to exercise [96]; it just seems that they, and allied health providers, need the right conditions to allow them to thrive.

## Strengths and limitations

This study has several strengths. First, the BELL trial was a replication of a successful community-based initiative. Healthy ageing is a high-priority focus of clinical practice, and results of the trial were compelling. The findings of this study increase our confidence that a similar community-based program, to promote healthy ageing, is likely to succeed. Second, appropriate procedures and practices were used to collect and analyse the data, sufficient to support the conclusions. Third, the findings are sincere and credible. The results extend our understanding of engaging with older adults in structured programs of group exercise, and challenge many preconceived notions of ageing, which may presently be a barrier for healthcare providers.

Despite the strengths of this study, it is not without limitations. Firstly, although the well-established relationship between the examiner and participants throughout the intervention and the present study, facilitated open and frank discussion, bias may have positively skewed participants’ responses. Secondly, the researcher’s role in knowledge production is at the heart of reflexive thematic analysis; themes evoke participants’ voices, but ultimately tell the researcher’s story about the data, which is a subjective interpretation developed with theoretical assumptions, which have been made through the lens of a particular social, cultural, disciplinary, and ideological position. Thirdly, the degree to which individual personality may have influenced the results of this study, are unknown [97]. Finally, the participants represented only one ethnic group: older Caucasian Australians. The likelihood however that these results may be transferable to other insufficiently active community-living older adults, is enhanced by data and thematic saturation, and the heterogenous representation of physical capacity, exercise experience, and health conditions represented within the group.

## Conclusions

The experiences of participants in the BELL trial, reveal the efficacy and perceived health benefits associated with group-based hardstyle kettlebell training with peers of a similar age, and highlight some of the challenges of delivering a group-based kettlebell program that meets the diverse needs of older adults. Group training provided a valued opportunity for participants to build a new social support network, which greatly contributed to the participants’ enjoyment of the training and high rate of engagement, with the instructor likely to be influential in optimising outcomes. Age, training status, sex, and health condition did not negatively impact engagement or participation in the training. Training performance for participants living with a chronic health condition or persistent pain, challenged their self-schema, often disconfirming maladaptive beliefs and behaviours. Participants’ experiences in this large-scale replication of a successful clinic-based group-exercise program, indicate that a pragmatic approach to hardstyle kettlebell training may be an effective means of engaging insufficiently active older adults in regular resistance-based exercise in the community, with minimal barriers to entry. These results corroborated earlier studies highlighting the value of the social features of group-exercise, in fostering a supportive social environment to optimise participants’ health and wellbeing. Further research is warranted to optimise program design and delivery within community settings, to test subgroups of older adults in a clinical framework, and to identify strategies which support long-term engagement. The findings of this study, in conjunction with the results of the BELL trial, provide sufficient evidence to support a recommendation that group kettlebell training initiatives, delivered by appropriately trained individuals, should be used to promote healthy ageing in the community. 

## Supplementary Information

**Additional file 1.** Survey Monkey Questionnaire_your kettlebell experience.

**Additional file 2** Survey Monkey Questionnaire_positive and beneficial effects.

**Additional file 3.** Survey Monkey Questionnaire_negative and undesirable effects.

## Data Availability

All data generated or analysed during this study are included in this published article. Bond University Human Research Ethics Committee has not provided authorisation for interview transcripts to be made available.
