# Supplementary material for: “If somebody had told me I’d feel like I do now, I wouldn’t have believed them…” older adults’ experiences of the BELL trial: a qualitative study
Source: BMC Geriatr. 2022 Jun 3;22:481. doi: 10.1186/s12877-022-03174-5 (PMC9164186; doi:10.1186/s12877-022-03174-5)
Supplement: Supplementary file 3 — Additional file 3. Survey Monkey Questionnaire_negative and undesirable effects. [file 12877_2022_3174_MOESM3_ESM.pdf]

Older adults' experiences of supervised hardstyle kettlebell training: A qualitative study from the BELL pragmatic controlled trial

SurveyMonkey questionnaire: *Your kettlebell experience*

**How strongly do you agree that the following were motivators for wanting to participate in the trial?**	Strongly agree	Disagree	Neither agree nor disagree	Agree	Strongly agree
To lose weight	○	○	○	○	○
To increase physical activity	○	○	○	○	○
To try something new	○	○	○	○	○
To keep independent and healthy	○	○	○	○	○
Having a reason to leave your home	○	○	○	○	○
To meet new people	○	○	○	○	○
To have a weekly routine	○	○	○	○	○
Recommended by friend or family member	○	○	○	○	○
To support someone else	○	○	○	○	○
To challenge yourself	○	○	○	○	○
You were previously very physically active	○	○	○	○	○
To prevent a chronic health condition e.g., heart disease	○	○	○	○	○
To be involved in a university research project	○	○	○	○	○
To 'give back' and help the community	○	○	○	○	○
Wanting to learn more about healthy ageing	○	○	○	○	○

**How strongly do you agree with the following statements?**	Strongly agree	Disagree	Neither agree nor disagree	Agree	Strongly agree
I enjoyed training with kettlebells	○	○	○	○	○
Kettlebell training has improved my physical health and/or capacity	○	○	○	○	○
Kettlebell training has improved my mental health and/or sense of wellbeing	○	○	○	○	○
I feel confident that I can train at home on my own with a kettlebell	○	○	○	○	○
I will continue using a kettlebell for exercise at home after the trial	○	○	○	○	○
What I've physically able to achieve exceeded my expectations	○	○	○	○	○
Regular physical activity or sport has played an important role in my life	○	○	○	○	○
I enjoyed exercising at home	○	○	○	○	○
The extra home exercises were helpful	○	○	○	○	○

How important were the following to your overall enjoyment of the training	Unimportant	Somewhat important	Moderately important	Very important	Extremely important
Variety of exercises	○	○	○	○	○
The instructor's personality	○	○	○	○	○
Social interaction and connecting with others	○	○	○	○	○
Exercising as a group	○	○	○	○	○
Having a weekly routine and structure	○	○	○	○	○
The format of the training sessions	○	○	○	○	○
Having set goals and reaching targets	○	○	○	○	○
Other (please specify):					

How aware have you been of the following	Not at all aware	Slightly aware	Moderately aware	Very aware	Extremely aware
Having a stronger and more enduring grip	○	○	○	○	○
Feeling less 'puffed' when walking quickly or ascending stairs	○	○	○	○	○
Having stronger legs	○	○	○	○	○
Being able to rise from a chair or seat quicker with less difficulty	○	○	○	○	○
Feeling less generalised physical fatigue	○	○	○	○	○
Physical changes i.e., less body fat or more muscle tone	○	○	○	○	○
Getting down to the ground and back up again with less difficulty	○	○	○	○	○
Lifting heavier objects around the home	○	○	○	○	○
Feeling less concerned about lifting or manipulating heavy objects	○	○	○	○	○
Feeling steadier and more balanced on your feet	○	○	○	○	○
Feeling more disciplined having a regular routine of exercise	○	○	○	○	○
Improvements in a chronic health condition such as back or knee conditions	○	○	○	○	○
Weight loss	○	○	○	○	○
Improved sleep	○	○	○	○	○
More energy	○	○	○	○	○
Feeling more cheerful or happy	○	○	○	○	○
Feeling more positive or encouraged	○	○	○	○	○
Improved wellbeing	○	○	○	○	○
Feeling a sense of achievement	○	○	○	○	○

Has your physical performance in the trial exceeded your prior expectations?

When you trained at home, did you train alone or was someone there to help or train with you?

During the trial, did you show or teach someone else how to use a kettlebell?

Have family or friends expressed interest in your participation in the trial and/or you training with a kettlebell? (If 'Yes', please explain:)

If the kettle bell training could have been designed and delivered just for you, what would it have looked like i.e., how would it have been different?
